# Benzyl­tributyl­ammonium 4-hydroxy­naphthalene-2-sulfonate

**DOI:** 10.1107/S1600536809000178

**Published:** 2009-01-17

**Authors:** Kazuya Uta, Jin Mizuguchi

**Affiliations:** a Department of Applied Physics, Graduate School of Engineering, Yokohama National University, 79-5 Tokiwadai, Hodogaya-ku, 240-8501 Yokohama, Japan

## Abstract

The title compound, C_19_H_34_N^+^·C_10_H_7_O_4_S^−^, is a charge-control agent used for toners in electrophotography. In the crystal structure, centrosymmetric anions associate through O—H⋯O hydrogen bonds formed between the O—H group of one anion and the sulfonate O atom of a neighbor. The components of the dimer are offset with respect to each other so that the separation between the two parallel naphthalene skeletons is about 1.6 Å. The ethyl residues of two of the butyl groups are disordered and were modelled over two postions (site occupancies = 0.33/0.67 and 0.34/0.66).

## Related literature

For the function of charge-control agents, see: Nash *et al.* (2001 ▶). For the structures of benzyl­tributyl­ammonium 4-hydroxy­naphthalene-1-sulfonate and benzyl­tributyl­ammonium 6-hydroxy­naphthalene-2-sulfonate, see: Mizuguchi *et al.* (2007 ▶) and Uta *et al.* (2009 ▶), respectively.
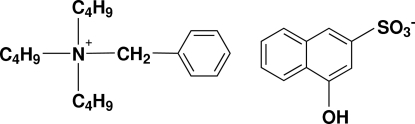


## Experimental

### 

#### Crystal data

C_19_H_34_N^+^·C_10_H_7_O_4_S^−^
*M* *_r_* = 499.70
Monoclinic, 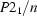

*a* = 11.2676 (11) Å
*b* = 12.4528 (12) Å
*c* = 20.549 (2) Å
β = 101.628 (7)°
*V* = 2824.1 (5) Å^3^
*Z* = 4
Cu *K*α radiation
μ = 1.27 mm^−1^
*T* = 296.1 K
0.50 × 0.35 × 0.35 mm

#### Data collection

Rigaku R-AXIS RAPID diffractometer
Absorption correction: multi-scan (*ABSCOR*; Higashi, 1995 ▶) *T*_min_ = 0.625, *T*_max_ = 0.640
25962 measured reflections
4907 independent reflections
4254 reflections with *F* ^2^ > 2σ(*F* ^2^)
*R*_int_ = 0.033

#### Refinement

*R*[*F* ^2^ > 2σ(*F* ^2^)] = 0.082
*wR*(*F* ^2^) = 0.221
*S* = 1.08
4907 reflections
347 parameters
H-atom parameters constrained
Δρ_max_ = 0.67 e Å^−3^
Δρ_min_ = −0.97 e Å^−3^

### 

Data collection: *PROCESS-AUTO* (Rigaku, 1998 ▶); cell refinement: *PROCESS-AUTO*; data reduction: *CrystalStructure* (Rigaku/MSC, 2006 ▶); program(s) used to solve structure: *SIR2002* (Burla *et al.*, 2003 ▶); program(s) used to refine structure: *SHELXL97* (Sheldrick, 2008 ▶); molecular graphics: *ORTEPIII* (Burnett & Johnson, 1996 ▶); software used to prepare material for publication: *CrystalStructure*.

## Supplementary Material

Crystal structure: contains datablocks global, I. DOI: 10.1107/S1600536809000178/tk2353sup1.cif

Structure factors: contains datablocks I. DOI: 10.1107/S1600536809000178/tk2353Isup2.hkl

Additional supplementary materials:  crystallographic information; 3D view; checkCIF report

## Figures and Tables

**Table 1:** Hydrogen-bond geometry (Å, °)

*D*—H⋯*A*	*D*—H	H⋯*A*	*D*⋯*A*	*D*—H⋯*A*
O4—H4*O*⋯O2^i^	0.82	1.90	2.706 (2)	167

Symmetry code: (i) 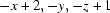.

**Fig. 1.**
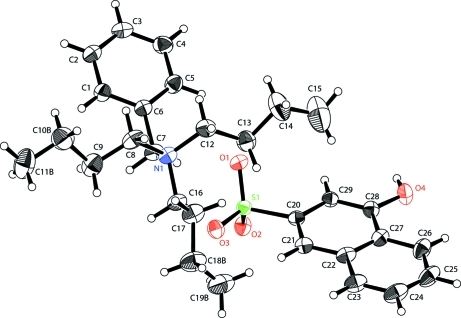
The asymmetric unit of (I), showing 30% probability displacement ellipsoids and only the major components of the disordered residues.

**Fig. 2.**
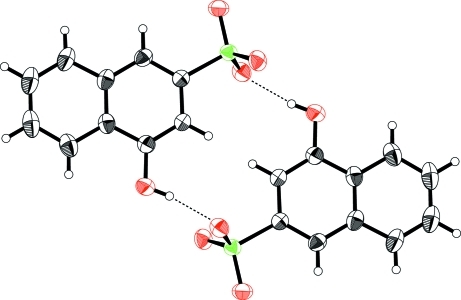
View of the hydrogen bonded anion dimer in (I). The O—-H···O hydrogen bonds are shown as dashed lines.

**Fig. 3.**
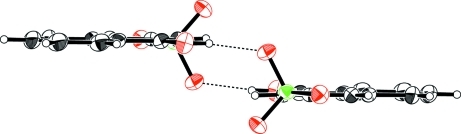
Side-on view of the hydrogen bonded anion dimer in (I) highlighting the step-like topology. The O—-H···O hydrogen bonds are shown as dashed lines.

## supplementary crystallographic information

### Comment

Compound (I) is a charge-control-agent used for toners in electrophotography. The background of the present study has been set out in a previous contribution (Uta *et al.*, 2009). We have previously investigated the crystal structures of two isomers of (I) in connection with the mechanism of their high thermal stability, namely benzyltributylammonium 4-hydroxynaphthalene-1-sulfonate (Mizuguchi *et al.*, 2007) and benzyltributylammonium 6-hydroxynaphthalene-2-sulfonate (Uta *et al.*, 2009). The anions in both isomers are found to form chains *via* O—H···O hydrogen bonds formed between the O—H group of one anion and the sulfonic-O atom of neighbors. The presence of these hydrogen-bonded networks ensures the high thermal stability of these compounds as characterized by their high melting points of 462 K and 433 K, respectively. This paper describes a variation to the above in that the O—H···O hydrogen bonding occurs between isolated pairs of molecules.

The asymmetric unit of (I) comprises a benzyltributylammonium cation and a 4-hydroxynaphthalene-2-sulfonate anion, Fig. 1. Centrosymmetrically related anions associate *via* O—H···O hydrogen bonds, Fig. 2 and Table 1, to form a 14-membered ring. As highlighted in the side-on view of Fig. 3, a step topology characterizes the dimeric unit so that the distance between the parallel naphthalene skeletons is about 1.6 Å. The hydrogen-bonded dimer in (I) is found to enhance the thermal stability as seen in the melting point of 451 K.

### Experimental

Compound (I) was obtained from Orient Chemical Industries, Ltd. and was recrystallized from a dichloromethane solution. After 48 h, a number of colorless crystals were obtained in the form of blocks.

### Refinement

Each of the two pairs of butyl-C10 & C11 and C18 & C19 atoms were found to be disordered over two sites. From anisotropic refinement, the site occupancies for the C10A/C10B and C11A/C11B pairs were fixed at 0.33 and 0.67, respectively, whereas those for C18A/C18B and C19A/C19B were fixed at 0.34 and 0.66, respectively. All H atoms were placed in their geometrically idealized positions and constrained to ride on their parent atoms, with C—H = 0.93 Å (aromatic), 0.96 Å (methyl), or 0.97 Å (methylene), and O—H = 0.82 Å, and with *U*_iso_(H) = 1.2-1.5*U*_eq_(parent atom).

### Crystal data

**Table:**

C_19_H_34_N^+^·C_10_H_7_O_4_S^−^	*F*(000) = 1080
*M**_r_* = 499.70	*D*_x_ = 1.175 Mg m^−^^3^
Monoclinic, *P*2_1_/*n*	Cu *K*α radiation, λ = 1.54187 Å
Hall symbol: -P 2yn	Cell parameters from 20030 reflections
*a* = 11.2676 (11) Å	θ = 3.5–68.2°
*b* = 12.4528 (12) Å	µ = 1.27 mm^−^^1^
*c* = 20.549 (2) Å	*T* = 296 K
β = 101.628 (7)°	Block, colorless
*V* = 2824.1 (5) Å^3^	0.50 × 0.35 × 0.35 mm
*Z* = 4	

### Data collection

**Table:**

Rigaku R-AXIS RAPID diffractometer	4254 reflections with *F*^2^ > 2σ(*F*^2^)
Detector resolution: 10.00 pixels mm^-1^	*R*_int_ = 0.033
ω scans	θ_max_ = 68.2°
Absorption correction: multi-scan (*ABSCOR*; Higashi, 1995)	*h* = −13→13
*T*_min_ = 0.625, *T*_max_ = 0.640	*k* = −15→15
25962 measured reflections	*l* = −24→24
4907 independent reflections	

### Refinement

**Table:**

Refinement on *F*^2^	H-atom parameters constrained
*R*[*F*^2^ > 2σ(*F*^2^)] = 0.082	*w* = 1/[σ^2^(*F*_o_^2^) + (0.1354*P*)^2^ + 1.1699*P*] where *P* = (*F*_o_^2^ + 2*F*_c_^2^)/3
*wR*(*F*^2^) = 0.221	(Δ/σ)_max_ < 0.001
*S* = 1.08	Δρ_max_ = 0.67 e Å^−^^3^
4907 reflections	Δρ_min_ = −0.97 e Å^−^^3^
347 parameters	

### Special details

**Table:**

Geometry. All e.s.d.'s (except the e.s.d. in the dihedral angle between two l.s. planes) are estimated using the full covariance matrix. The cell e.s.d.'s are taken into account individually in the estimation of e.s.d.'s in distances, angles and torsion angles; correlations between e.s.d.'s in cell parameters are only used when they are defined by crystal symmetry. An approximate (isotropic) treatment of cell e.s.d.'s is used for estimating e.s.d.'s involving l.s. planes.
Refinement. Refinement of *F*^2^ against ALL reflections. The weighted *R*-factor *wR* and goodness of fit *S* are based on *F*^2^, conventional *R*-factors *R* are based on *F*, with *F* set to zero for negative *F*^2^. The threshold expression of *F*^2^ > σ(*F*^2^) is used only for calculating *R*-factors(gt) *etc*. and is not relevant to the choice of reflections for refinement. *R*-factors based on *F*^2^ are statistically about twice as large as those based on *F*, and *R*-factors based on ALL data will be even larger.

### Fractional atomic coordinates and isotropic or equivalent isotropic displacement parameters (Å^2^)

**Table:**

	*x*	*y*	*z*	*U*_iso_*/*U*_eq_	Occ. (<1)
S1	0.91846 (5)	0.00482 (5)	0.63573 (3)	0.0505 (2)	
O1	0.80247 (16)	0.02686 (17)	0.59362 (10)	0.0661 (5)	
O2	0.97467 (17)	−0.09153 (14)	0.61559 (10)	0.0630 (4)	
O3	0.91597 (19)	0.00507 (16)	0.70580 (10)	0.0672 (5)	
O4	1.1388 (2)	0.22083 (17)	0.48351 (10)	0.0714 (5)	
N1	0.63389 (19)	0.16264 (17)	0.75442 (12)	0.0573 (5)	
C1	0.4036 (2)	−0.0072 (2)	0.68571 (16)	0.0625 (7)	
C2	0.3010 (2)	−0.0304 (2)	0.63847 (17)	0.0691 (7)	
C3	0.2984 (2)	−0.0091 (2)	0.57251 (18)	0.0703 (8)	
C4	0.3972 (3)	0.0358 (2)	0.55390 (16)	0.0737 (8)	
C5	0.5013 (2)	0.0591 (2)	0.60108 (15)	0.0677 (7)	
C6	0.5045 (2)	0.0377 (2)	0.66767 (14)	0.0541 (6)	
C7	0.6212 (2)	0.0541 (2)	0.71838 (14)	0.0581 (6)	
C8	0.5322 (2)	0.1792 (2)	0.79266 (16)	0.0734 (8)	
C9	0.53000 (17)	0.1034 (3)	0.85017 (16)	0.0945 (10)	
C10A	0.4160 (3)	0.0773 (3)	0.8763 (3)	0.105 (2)	0.33
C10B	0.4118 (3)	0.1309 (3)	0.8716 (2)	0.1068 (19)	0.67
C11A	0.3668 (12)	0.1744 (7)	0.9023 (8)	0.133 (2)	0.33
C11B	0.4095 (7)	0.0758 (6)	0.9349 (3)	0.133 (2)	0.67
C12	0.6247 (2)	0.2546 (2)	0.70588 (16)	0.0695 (7)	
C13	0.7250 (3)	0.2623 (2)	0.66732 (16)	0.0794 (9)	
C14	0.6911 (2)	0.3473 (3)	0.6135 (2)	0.1264 (16)	
C15	0.7786 (6)	0.3542 (6)	0.5691 (3)	0.200 (3)	
C16	0.7576 (2)	0.1590 (2)	0.80101 (15)	0.0625 (7)	
C17	0.7946 (2)	0.2602 (2)	0.84116 (16)	0.0797 (8)	
C18A	0.9264 (3)	0.2545 (3)	0.8776 (4)	0.097 (3)	0.34
C18B	0.9066 (4)	0.2397 (6)	0.8951 (2)	0.107 (2)	0.66
C19A	0.9500 (12)	0.1705 (8)	0.9291 (5)	0.1192 (19)	0.34
C19B	1.0137 (5)	0.2081 (6)	0.8686 (3)	0.1192 (19)	0.66
C20	1.0176 (2)	0.10968 (19)	0.62246 (12)	0.0490 (5)	
C21	1.0829 (2)	0.1662 (2)	0.67460 (14)	0.0567 (6)	
C22	1.1668 (2)	0.2447 (2)	0.66307 (15)	0.0592 (6)	
C23	1.2369 (2)	0.3057 (2)	0.71564 (18)	0.0758 (8)	
C24	1.3190 (3)	0.3785 (2)	0.7044 (2)	0.0871 (10)	
C25	1.3381 (2)	0.3946 (2)	0.6407 (2)	0.0880 (11)	
C26	1.2734 (2)	0.3379 (2)	0.58762 (19)	0.0751 (8)	
C27	1.1857 (2)	0.2624 (2)	0.59803 (15)	0.0578 (6)	
C28	1.1160 (2)	0.2017 (2)	0.54510 (13)	0.0543 (6)	
C29	1.0324 (2)	0.1288 (2)	0.55687 (12)	0.0504 (5)	
H1	0.4048	−0.0221	0.7302	0.075*	
H2	0.2337	−0.0604	0.6513	0.083*	
H3	0.2296	−0.0251	0.5406	0.084*	
H4	0.3949	0.0510	0.5093	0.088*	
H4O	1.0955	0.1822	0.4563	0.086*	
H5	0.5684	0.0889	0.5880	0.081*	
H7A	0.6877	0.0472	0.6953	0.073*	
H7B	0.6276	−0.0019	0.7507	0.073*	
H8A	0.5377	0.2521	0.8097	0.088*	
H8B	0.4554	0.1729	0.7615	0.088*	
H9A	0.5886	0.1310	0.8876	0.113*	0.33
H9B	0.5617	0.0353	0.8382	0.113*	0.33
H9C	0.5990	0.1156	0.8861	0.113*	0.67
H9D	0.5304	0.0291	0.8360	0.113*	0.67
H10A	0.3554	0.0471	0.8407	0.125*	0.33
H10B	0.4349	0.0241	0.9113	0.125*	0.33
H10C	0.4060	0.2079	0.8773	0.128*	0.67
H10D	0.3435	0.1079	0.8379	0.128*	0.67
H11A	0.2989	0.1551	0.9216	0.200*	0.33
H11B	0.3411	0.2244	0.8667	0.200*	0.33
H11C	0.4284	0.2069	0.9356	0.200*	0.33
H11D	0.4257	0.0007	0.9305	0.200*	0.67
H11E	0.3311	0.0845	0.9458	0.200*	0.67
H11F	0.4701	0.1062	0.9695	0.200*	0.67
H12A	0.5483	0.2485	0.6745	0.083*	
H12B	0.6227	0.3211	0.7302	0.083*	
H13A	0.7360	0.1934	0.6473	0.095*	
H13B	0.8004	0.2817	0.6969	0.095*	
H14A	0.6116	0.3310	0.5872	0.152*	
H14B	0.6863	0.4167	0.6344	0.152*	
H15A	0.8566	0.3743	0.5944	0.300*	
H15B	0.7518	0.4071	0.5354	0.300*	
H15C	0.7845	0.2857	0.5486	0.300*	
H16A	0.7581	0.0996	0.8316	0.075*	
H16B	0.8183	0.1439	0.7748	0.075*	
H17A	0.7429	0.2696	0.8732	0.096*	0.34
H17B	0.7836	0.3218	0.8117	0.096*	0.34
H17C	0.7284	0.2837	0.8614	0.096*	0.66
H17D	0.8116	0.3168	0.8119	0.096*	0.66
H18A	0.9770	0.2412	0.8454	0.116*	0.34
H18B	0.9495	0.3235	0.8981	0.116*	0.34
H18C	0.9255	0.3044	0.9215	0.129*	0.66
H18D	0.8886	0.1834	0.9242	0.129*	0.66
H19A	0.8759	0.1337	0.9309	0.179*	0.34
H19B	0.9818	0.2029	0.9715	0.179*	0.34
H19C	1.0079	0.1202	0.9186	0.179*	0.34
H19D	0.9994	0.1395	0.8471	0.179*	0.66
H19E	1.0830	0.2034	0.9043	0.179*	0.66
H19F	1.0286	0.2608	0.8370	0.179*	0.66
H21	1.0722	0.1530	0.7176	0.068*	
H23	1.2258	0.2951	0.7588	0.091*	
H24	1.3629	0.4180	0.7395	0.104*	
H25	1.3955	0.4446	0.6336	0.106*	
H26	1.2875	0.3493	0.5451	0.090*	
H29	0.9851	0.0918	0.5217	0.060*	

### Atomic displacement parameters (Å^2^)

**Table:**

	*U*^11^	*U*^22^	*U*^33^	*U*^12^	*U*^13^	*U*^23^
S1	0.0471 (3)	0.0556 (3)	0.0498 (4)	0.0019 (2)	0.0121 (2)	0.0009 (2)
O1	0.0447 (9)	0.0798 (12)	0.0700 (13)	−0.0022 (8)	0.0026 (8)	0.0053 (9)
O2	0.0680 (11)	0.0481 (9)	0.0778 (12)	0.0001 (8)	0.0264 (9)	−0.0020 (8)
O3	0.0714 (12)	0.0808 (13)	0.0529 (12)	−0.0005 (9)	0.0208 (9)	0.0039 (8)
O4	0.0883 (13)	0.0682 (12)	0.0624 (12)	−0.0186 (10)	0.0261 (10)	0.0013 (9)
N1	0.0523 (11)	0.0544 (11)	0.0650 (14)	0.0038 (9)	0.0118 (10)	−0.0133 (9)
C1	0.0623 (16)	0.0628 (16)	0.0629 (18)	−0.0047 (12)	0.0140 (14)	−0.0025 (12)
C2	0.0529 (14)	0.0727 (17)	0.080 (2)	−0.0037 (13)	0.0091 (14)	−0.0040 (15)
C3	0.0572 (16)	0.0753 (19)	0.072 (2)	0.0098 (13)	−0.0029 (14)	−0.0090 (14)
C4	0.0740 (18)	0.089 (2)	0.0562 (18)	0.0045 (16)	0.0088 (14)	−0.0033 (15)
C5	0.0629 (15)	0.0783 (19)	0.0640 (18)	−0.0043 (14)	0.0181 (13)	−0.0072 (14)
C6	0.0522 (13)	0.0503 (13)	0.0597 (16)	0.0044 (10)	0.0109 (11)	−0.0074 (11)
C7	0.0539 (13)	0.0514 (13)	0.0659 (17)	0.0030 (11)	0.0050 (12)	−0.0156 (11)
C8	0.0551 (14)	0.082 (2)	0.085 (2)	0.0037 (14)	0.0181 (14)	−0.0301 (16)
C9	0.091 (2)	0.118 (2)	0.079 (2)	−0.016 (2)	0.0288 (18)	−0.021 (2)
C10A	0.108 (5)	0.131 (6)	0.089 (5)	−0.011 (5)	0.051 (4)	0.013 (5)
C10B	0.107 (3)	0.107 (4)	0.107 (4)	0.000 (3)	0.022 (3)	0.000 (3)
C11A	0.136 (4)	0.150 (5)	0.133 (4)	−0.034 (4)	0.072 (3)	−0.023 (3)
C11B	0.136 (4)	0.150 (5)	0.133 (4)	−0.034 (4)	0.072 (3)	−0.023 (3)
C12	0.0718 (17)	0.0527 (15)	0.080 (2)	0.0071 (12)	0.0058 (15)	−0.0059 (13)
C13	0.088 (2)	0.0681 (18)	0.083 (2)	−0.0052 (16)	0.0189 (18)	−0.0014 (15)
C14	0.180 (4)	0.087 (2)	0.124 (3)	0.009 (3)	0.057 (3)	0.023 (2)
C15	0.257 (8)	0.173 (6)	0.199 (6)	−0.004 (6)	0.115 (6)	0.052 (5)
C16	0.0512 (13)	0.0635 (15)	0.0697 (18)	0.0009 (11)	0.0051 (12)	−0.0117 (12)
C17	0.0754 (18)	0.0747 (18)	0.085 (2)	−0.0020 (15)	0.0072 (15)	−0.0266 (15)
C18A	0.082 (4)	0.091 (5)	0.106 (6)	−0.013 (5)	−0.006 (5)	−0.035 (4)
C18B	0.089 (3)	0.110 (4)	0.108 (4)	−0.007 (3)	−0.013 (2)	−0.025 (3)
C19A	0.082 (2)	0.115 (4)	0.146 (5)	0.003 (3)	−0.010 (2)	−0.008 (3)
C19B	0.082 (2)	0.115 (4)	0.146 (5)	0.003 (3)	−0.010 (2)	−0.008 (3)
C20	0.0459 (11)	0.0502 (12)	0.0502 (14)	0.0061 (9)	0.0079 (10)	−0.0018 (10)
C21	0.0557 (13)	0.0608 (14)	0.0530 (15)	0.0054 (11)	0.0096 (11)	−0.0075 (11)
C22	0.0514 (13)	0.0541 (14)	0.0691 (18)	0.0071 (11)	0.0045 (12)	−0.0155 (11)
C23	0.0674 (17)	0.0699 (18)	0.083 (2)	0.0005 (14)	−0.0009 (15)	−0.0261 (15)
C24	0.0656 (18)	0.071 (2)	0.117 (3)	−0.0029 (15)	0.0002 (19)	−0.0342 (19)
C25	0.0600 (17)	0.0616 (18)	0.140 (3)	−0.0112 (14)	0.015 (2)	−0.022 (2)
C26	0.0644 (16)	0.0560 (16)	0.109 (2)	−0.0063 (13)	0.0266 (17)	−0.0067 (15)
C27	0.0514 (13)	0.0462 (12)	0.0762 (18)	0.0008 (10)	0.0137 (12)	−0.0077 (11)
C28	0.0576 (13)	0.0478 (12)	0.0586 (15)	0.0018 (10)	0.0144 (11)	−0.0004 (10)
C29	0.0512 (12)	0.0498 (12)	0.0483 (14)	0.0005 (10)	0.0057 (10)	−0.0033 (10)

### Geometric parameters (Å, °)

**Table:**

S1—O1	1.4409 (17)	C7—H7A	0.968
S1—O2	1.4552 (19)	C7—H7B	0.955
S1—O3	1.446 (2)	C8—H8A	0.970
S1—C20	1.775 (2)	C8—H8B	0.970
O4—C28	1.362 (3)	C9—H9A	0.970
N1—C7	1.535 (3)	C9—H9B	0.970
N1—C8	1.529 (4)	C10A—H10A	0.970
N1—C12	1.508 (3)	C10A—H10B	0.970
N1—C16	1.524 (3)	C10B—H10C	0.970
C1—C2	1.382 (4)	C10B—H10D	0.970
C1—C6	1.383 (4)	C11A—H11A	0.960
C2—C3	1.376 (5)	C11A—H11B	0.960
C3—C4	1.367 (4)	C11A—H11C	0.960
C4—C5	1.393 (4)	C11B—H11D	0.960
C5—C6	1.387 (4)	C11B—H11E	0.960
C6—C7	1.518 (3)	C11B—H11F	0.960
C8—C9	1.517 (4)	C12—H12A	0.970
C9—C10A	1.523 (5)	C12—H12B	0.970
C9—C10B	1.524 (5)	C13—H13A	0.970
C10A—C11A	1.475 (13)	C13—H13B	0.970
C10B—C11B	1.475 (8)	C14—H14A	0.970
C12—C13	1.508 (5)	C14—H14B	0.970
C13—C14	1.523 (4)	C15—H15A	0.960
C14—C15	1.474 (8)	C15—H15B	0.960
C16—C17	1.518 (4)	C15—H15C	0.960
C17—C18A	1.524 (5)	C16—H16A	0.970
C17—C18B	1.524 (5)	C16—H16B	0.970
C18A—C19A	1.475 (13)	C17—H17A	0.970
C18B—C19B	1.475 (8)	C17—H17B	0.970
C20—C21	1.367 (3)	C18A—H18A	0.970
C20—C29	1.411 (3)	C18A—H18B	0.970
C21—C22	1.412 (3)	C18B—H18C	0.970
C22—C23	1.423 (4)	C18B—H18D	0.970
C22—C27	1.413 (4)	C19A—H19A	0.960
C23—C24	1.349 (5)	C19A—H19B	0.960
C24—C25	1.383 (6)	C19A—H19C	0.960
C25—C26	1.378 (5)	C19B—H19D	0.960
C26—C27	1.411 (4)	C19B—H19E	0.960
C27—C28	1.424 (3)	C19B—H19F	0.960
C28—C29	1.364 (3)	C21—H21	0.930
O4—H4O	0.820	C23—H23	0.930
C1—H1	0.930	C24—H24	0.930
C2—H2	0.930	C25—H25	0.930
C3—H3	0.930	C26—H26	0.930
C4—H4	0.930	C29—H29	0.930
C5—H5	0.930		
			
O2···O4^i^	2.706 (2)	C5···H18B^iv^	2.985
O4···O2^i^	2.706 (2)	C7···H9D	2.826
S1···H4O^i^	2.984	C8···H9C	2.073
O1···H3^ii^	2.710	C8···H9D	2.073
O1···H4^ii^	2.910	C8···H17C	2.706
O1···H5	2.728	C9···H9C	0.970
O1···H7A	2.681	C9···H9D	0.970
O1···H13A	2.532	C10B···H9C	2.079
O2···H2^iii^	2.889	C10B···H9D	2.079
O2···H4O^i^	1.900	C11B···H15B^vii^	2.991
O2···H8A^iv^	2.501	C11B···H9C	2.585
O2···H10C^iv^	2.826	C11B···H9D	2.728
O2···H29^i^	2.945	C12···H17D	2.816
O2···H17C^iv^	2.882	C15···H11E^vi^	2.822
O3···H7A	2.590	C16···H9C	2.792
O3···H12B^iv^	2.720	C16···H17C	2.056
O3···H16B	2.615	C16···H17D	2.056
O3···H24^v^	2.742	C17···H17C	0.970
O4···H9A^vi^	2.676	C17···H17D	0.970
O4···H11C^vi^	2.542	C18B···H17C	2.061
O4···H11F^vi^	2.848	C18B···H17D	2.061
O4···H17A^vi^	2.757	C19B···H17D	2.703
O4···H9C^vi^	2.829	C23···H8B^iii^	2.958
O4···H17C^vi^	2.887	C24···H10A^viii^	2.897
N1···H9C	2.871	C25···H10A^viii^	2.973
N1···H9D	2.779	C25···H12A^iii^	2.956
N1···H17C	2.702	C26···H11D^viii^	2.991
N1···H17D	2.852	C28···H11C^vi^	2.985
			
O1—S1—O2	112.35 (11)	C9—C10B—H10C	109.9
O1—S1—O3	113.60 (12)	C9—C10B—H10D	109.9
O1—S1—C20	106.88 (11)	C11B—C10B—H10C	109.9
O2—S1—O3	112.45 (11)	C11B—C10B—H10D	109.9
O2—S1—C20	103.85 (11)	H10C—C10B—H10D	108.3
O3—S1—C20	106.89 (11)	C10A—C11A—H11A	109.5
C7—N1—C8	111.1 (2)	C10A—C11A—H11B	109.5
C7—N1—C12	111.2 (2)	C10A—C11A—H11C	109.5
C7—N1—C16	105.12 (18)	H11A—C11A—H11B	109.5
C8—N1—C12	106.2 (2)	H11A—C11A—H11C	109.5
C8—N1—C16	111.5 (2)	H11B—C11A—H11C	109.5
C12—N1—C16	111.7 (2)	C10B—C11B—H11D	109.5
C2—C1—C6	120.8 (3)	C10B—C11B—H11E	109.5
C1—C2—C3	120.0 (3)	C10B—C11B—H11F	109.5
C2—C3—C4	119.8 (2)	H11D—C11B—H11E	109.5
C3—C4—C5	120.6 (3)	H11D—C11B—H11F	109.5
C4—C5—C6	119.8 (2)	H11E—C11B—H11F	109.5
C1—C6—C5	118.9 (2)	N1—C12—H12A	108.3
C1—C6—C7	121.0 (2)	N1—C12—H12B	108.3
C5—C6—C7	119.9 (2)	C13—C12—H12A	108.3
N1—C7—C6	115.6 (2)	C13—C12—H12B	108.3
N1—C8—C9	116.6 (2)	H12A—C12—H12B	107.4
C8—C9—C10A	123.6 (2)	C12—C13—H13A	109.9
C8—C9—C10B	103.8 (2)	C12—C13—H13B	109.9
C9—C10B—C11B	108.8 (4)	C14—C13—H13A	109.9
N1—C12—C13	115.9 (2)	C14—C13—H13B	109.9
C12—C13—C14	108.7 (2)	H13A—C13—H13B	108.3
C13—C14—C15	112.9 (3)	C13—C14—H14A	109.0
N1—C16—C17	115.6 (2)	C13—C14—H14B	109.0
C16—C17—C18A	111.2 (3)	C15—C14—H14A	109.0
C16—C17—C18B	110.7 (3)	C15—C14—H14B	109.0
C17—C18A—C19A	113.8 (6)	H14A—C14—H14B	107.8
C17—C18B—C19B	113.3 (4)	C14—C15—H15A	109.5
S1—C20—C21	121.0 (2)	C14—C15—H15B	109.5
S1—C20—C29	118.05 (17)	C14—C15—H15C	109.5
C21—C20—C29	120.9 (2)	H15A—C15—H15B	109.5
C20—C21—C22	119.9 (2)	H15A—C15—H15C	109.5
C21—C22—C23	122.0 (2)	H15B—C15—H15C	109.5
C21—C22—C27	120.1 (2)	N1—C16—H16A	108.4
C23—C22—C27	117.9 (2)	N1—C16—H16B	108.4
C22—C23—C24	121.6 (3)	C17—C16—H16A	108.4
C23—C24—C25	120.2 (3)	C17—C16—H16B	108.4
C24—C25—C26	121.0 (3)	H16A—C16—H16B	107.4
C25—C26—C27	119.8 (3)	C16—C17—H17A	109.4
C22—C27—C26	119.4 (2)	C16—C17—H17B	109.4
C22—C27—C28	118.3 (2)	C16—C17—H17C	109.5
C26—C27—C28	122.2 (2)	C16—C17—H17D	109.5
O4—C28—C27	116.0 (2)	C18A—C17—H17A	109.4
O4—C28—C29	123.2 (2)	C18A—C17—H17B	109.4
C27—C28—C29	120.8 (2)	C18B—C17—H17C	109.5
C20—C29—C28	120.0 (2)	C18B—C17—H17D	109.5
C28—O4—H4O	109.5	H17A—C17—H17B	108.0
C2—C1—H1	119.6	H17C—C17—H17D	108.1
C6—C1—H1	119.6	C17—C18A—H18A	108.8
C1—C2—H2	120.0	C17—C18A—H18B	108.8
C3—C2—H2	120.0	C19A—C18A—H18A	108.8
C2—C3—H3	120.1	C19A—C18A—H18B	108.8
C4—C3—H3	120.1	H18A—C18A—H18B	107.7
C3—C4—H4	119.7	C17—C18B—H18C	108.9
C5—C4—H4	119.7	C17—C18B—H18D	108.9
C4—C5—H5	120.1	C19B—C18B—H18C	108.9
C6—C5—H5	120.1	C19B—C18B—H18D	108.9
N1—C7—H7A	108.0	H18C—C18B—H18D	107.7
N1—C7—H7B	108.6	C18A—C19A—H19A	109.5
C6—C7—H7A	107.5	C18A—C19A—H19B	109.5
C6—C7—H7B	108.3	C18A—C19A—H19C	109.5
H7A—C7—H7B	108.6	H19A—C19A—H19B	109.5
N1—C8—H8A	108.1	H19A—C19A—H19C	109.5
N1—C8—H8B	108.1	H19B—C19A—H19C	109.5
C9—C8—H8A	108.1	C18B—C19B—H19D	109.5
C9—C8—H8B	108.1	C18B—C19B—H19E	109.5
H8A—C8—H8B	107.3	C18B—C19B—H19F	109.5
C8—C9—H9A	106.4	H19D—C19B—H19E	109.5
C8—C9—H9B	106.4	H19D—C19B—H19F	109.5
C8—C9—H9C	111.0	H19E—C19B—H19F	109.5
C8—C9—H9D	111.0	C20—C21—H21	120.1
C10A—C9—H9A	106.4	C22—C21—H21	120.1
C10A—C9—H9B	106.4	C22—C23—H23	119.2
C10B—C9—H9C	111.0	C24—C23—H23	119.2
C10B—C9—H9D	111.0	C23—C24—H24	119.9
H9A—C9—H9B	106.5	C25—C24—H24	119.9
H9C—C9—H9D	109.0	C24—C25—H25	119.5
C9—C10A—H10A	109.4	C26—C25—H25	119.5
C9—C10A—H10B	109.4	C25—C26—H26	120.1
C11A—C10A—H10A	109.4	C27—C26—H26	120.1
C11A—C10A—H10B	109.4	C20—C29—H29	120.0
H10A—C10A—H10B	108.0	C28—C29—H29	120.0
			
O1—S1—C20—C21	127.8 (2)	C8—C9—C10A—C11A	−62.4 (8)
O1—S1—C20—C29	−55.8 (2)	C8—C9—C10B—C11B	−170.1 (4)
O2—S1—C20—C21	−113.2 (2)	N1—C12—C13—C14	171.0 (2)
O2—S1—C20—C29	63.2 (2)	C12—C13—C14—C15	−174.9 (3)
O3—S1—C20—C21	5.9 (2)	N1—C16—C17—C18A	−171.1 (3)
O3—S1—C20—C29	−177.75 (19)	N1—C16—C17—C18B	168.8 (3)
C7—N1—C8—C9	65.3 (2)	C16—C17—C18B—C19B	61.8 (6)
C8—N1—C7—C6	60.0 (3)	S1—C20—C21—C22	176.06 (19)
C7—N1—C12—C13	−65.4 (2)	S1—C20—C29—C28	−173.89 (19)
C12—N1—C7—C6	−58.1 (3)	C21—C20—C29—C28	2.5 (3)
C7—N1—C16—C17	177.8 (2)	C29—C20—C21—C22	−0.2 (3)
C16—N1—C7—C6	−179.2 (2)	C20—C21—C22—C23	179.96 (18)
C8—N1—C12—C13	173.6 (2)	C20—C21—C22—C27	−1.9 (3)
C12—N1—C8—C9	−173.6 (2)	C21—C22—C23—C24	178.3 (2)
C8—N1—C16—C17	−61.7 (3)	C21—C22—C27—C26	−177.4 (2)
C16—N1—C8—C9	−51.6 (3)	C21—C22—C27—C28	1.8 (3)
C12—N1—C16—C17	57.0 (3)	C23—C22—C27—C26	0.8 (3)
C16—N1—C12—C13	51.8 (3)	C23—C22—C27—C28	180.0 (2)
C2—C1—C6—C5	−0.2 (3)	C27—C22—C23—C24	0.1 (3)
C2—C1—C6—C7	−174.4 (2)	C22—C23—C24—C25	−0.8 (5)
C6—C1—C2—C3	0.2 (4)	C23—C24—C25—C26	0.6 (5)
C1—C2—C3—C4	−0.5 (4)	C24—C25—C26—C27	0.3 (4)
C2—C3—C4—C5	0.8 (5)	C25—C26—C27—C22	−1.0 (4)
C3—C4—C5—C6	−0.7 (5)	C25—C26—C27—C28	179.8 (2)
C4—C5—C6—C1	0.4 (4)	C22—C27—C28—O4	−179.2 (2)
C4—C5—C6—C7	174.7 (2)	C22—C27—C28—C29	0.5 (3)
C1—C6—C7—N1	−90.5 (3)	C26—C27—C28—O4	−0.1 (2)
C5—C6—C7—N1	95.4 (3)	C26—C27—C28—C29	179.6 (2)
N1—C8—C9—C10A	−155.4 (3)	O4—C28—C29—C20	177.1 (2)
N1—C8—C9—C10B	−173.1 (2)	C27—C28—C29—C20	−2.6 (3)

Symmetry codes: (i) −*x*+2, −*y*, −*z*+1; (ii) −*x*+1, −*y*, −*z*+1; (iii) *x*+1, *y*, *z*; (iv) −*x*+3/2, *y*−1/2, −*z*+3/2; (v) −*x*+5/2, *y*−1/2, −*z*+3/2; (vi) *x*+1/2, −*y*+1/2, *z*−1/2; (vii) *x*−1/2, −*y*+1/2, *z*+1/2; (viii) −*x*+3/2, *y*+1/2, −*z*+3/2.

### Hydrogen-bond geometry (Å, °)

**Table:**

*D*—H···*A*	*D*—H	H···*A*	*D*···*A*	*D*—H···*A*
O4—H4O···O2^i^	0.82	1.90	2.706 (2)	167

Symmetry codes: (i) −*x*+2, −*y*, −*z*+1.
